# Use of different food additives to control browning in fresh‐cut potatoes

**DOI:** 10.1002/fsn3.3714

**Published:** 2023-10-06

**Authors:** Guoqin Li, Xinxin Wang, Hongmei Zhu, Guifeng Li, Junjie Du, Xiaoqing Song, Erihemu

**Affiliations:** ^1^ School of Food Science Shanxi Normal University Taiyuan China; ^2^ Department of Life Modern College of Humanities and Sciences of Shanxi Normal University Linfen China; ^3^ School of Life Science Shanxi Normal University Taiyuan China

**Keywords:** ascorbic acid, hydrogen sulfide, L‐cysteine, nitric oxide, PPO and POD activities, total phenolic content

## Abstract

Fresh‐cut potato browning is a severe problem in the potato processing industry. Ascorbic acid, L‐cysteine, hydrogen sulfide (H_2_S), and nitric oxide (NO) have been reported to reduce the browning in fresh‐cut vegetables and fruits. We compared the effect of each food additive at its commonly used concentration on fresh‐cut potato browning in order to choose a highly efficient treatment and explore its mechanism. Fresh‐cut potato slices were immersed in 0.3 mmol L^−1^ ascorbic acid, 0.7 mmol L^−1^ L‐cysteine, 0.7 mmol L^−1^ H_2_S, or 2.0 mmol L^−1^ NO for 10 min and stored at 4°C until the measurements finished. Results showed that the ascorbic acid and L‐cysteine treatments showed less browning than the control treatment, while the H_2_S and NO treatments did not. Ascorbic acid increased total phenolic content, polyphenol oxidase (PPO) and peroxidase (POD) activities, while L‐cysteine decreased PPO and POD activities with no change in total phenolic content. In addition, these two treatments did not influence respiration rate, weight loss, or rot index. In conclusion, ascorbic acid (0.3 mmol L^−1^) and L‐cysteine (0.7 mmol L^−1^) can be valuable means to control fresh‐cut potato browning. Ascorbic acid inhibits the browning mainly by reducing quinones back to phenolic compounds, but L‐cysteine inhibits the browning mainly by decreasing PPO and POD activities.

## Graphical abstract

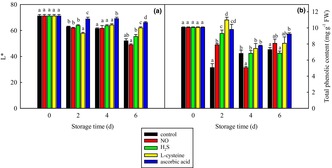

## 1 INTRODUCTION

Potato (*Solanum tuberosum* L.) is a widely spread vegetable, with 368 million tons produced in 2018 (FAOSTAT, 2020). Fresh‐cut potatoes have been accepted by consumers because of their convenience and high usable proportion (Wang et al., 2015). However, fresh‐cut potatoes tend to be browning during storage, which decreases quality and finally affects consumer purchase (Erihemu et al., 2021).

Enzymatic browning is mainly involved in the browning of fresh‐cut potatoes (Rashid et al., 2021). After the whole potato tuber is processed, the cell membrane is disrupted. Enzymes such as polyphenol oxidase (PPO) and peroxidase (POD) in cell cytoplasm contact with phenolics in the cell vacuole, and the enzymes oxidize the phenolics to be quinones which further form melanoid pigments (Liu et al., 2018).

Four food additives such as ascorbic acid, L‐cysteine, hydrogen sulfide (H_2_S), and nitric oxide (NO) have been commonly used to inhibit the browning of fresh‐cut vegetables and fruits. For example, dipping into ascorbic acid can reduce browning on fresh‐cut apples (Li et al., 2015) and pears (Zhu et al., 2009). L‐cysteine has been recently reported to inhibit browning in fresh‐cut apples and potatoes (Cerit et al., 2020; Erihemu et al., 2022). H_2_S and NO, two signal molecules involved in plants' defense mechanism, can inhibit browning of fresh‐cut carrots and apples (Chen et al., 2018; Huque et al., 2013; Zheng et al., 2016). However, it is not clear which food additive is more effective in inhibiting the browning of fresh‐cut potatoes.

Considering that the concentration of ascorbic acid, L‐cysteine, NO, and H_2_S applied to reduce the browning of fresh‐cut vegetables and fruits is different (Chen et al., 2020; Erihemu et al., 2022; Huque et al., 2013; Yu & Xie, 2019), we chose the commonly used concentration for each agent to reduce fresh‐cut potato slices' browning in this experiment. This study investigated the effects of ascorbic acid (0.3 mmol L^−1^), L‐cysteine (0.7 mmol L^−1^), H_2_S (0.7 mmol L^−1^), and NO (2 mmol L^−1^) on the browning, total phenolics content, and enzyme (PPO and POD) activities of fresh‐cut potato slices to explore a highly efficient treatment and its mechanism for inhibiting browning. The parameters such as rot index, respiration rate and weight loss were also measured to know different food additive effects on the quality of fresh‐cut potato slices, which can provide theoretical support for the potato processing industry.

## 2 MATERIALS AND METHODS

### 2.1 Materials

Fresh potato tubers (Jinshu 16) of the same size with no physical damage, insects, or diseases were purchased from Yaofeng Market, Linfen, Shanxi Province, China. We transported the potato tubers to a laboratory in half an hour and stored them at a low temperature (4°C) in darkness for use within 2 or 3 days. The potato tubers were manually peeled with a sharp knife, washed with tap water, and cut into 0.5‐cm‐thickness slices using a commercial cutting machine (LC‐Q01, Foshan Shunde Hantai Electric Appliance Co. Ltd). All the materials and equipment involved in the following treatments were first sterilized with a chlorine solution at 100 mg L^−1^.

Reagents used were as follows: ascorbic acid (Kermel, Tianjin Kermel Chemical Reagent Co. Ltd), L‐cysteine (Weiduomei, Guangdong Weiduomei Food Ingredients Co. Ltd), sodium nitroprusside (Kermel, Tianjin Kermel Chemical Regent Co. Ltd), sodium hydrosulfide (Energy Chemical, Saen Chemistry Technology Ltd Company), sodium hypochlorite (Kermel, Tianjin Kermel Chemical Regent Co. Ltd), phosphate buffer (Aladdin, Shanghai Aladdin Biochemical Technology Co. Ltd), gallic acid (Yuanye, Shanghai Yuanye Bio‐Technology Co. Ltd), Folin–Ciocalteu reagent (Yuanye, Shanghai Yuanye Bio‐Technology Co. Ltd), Na_2_CO_3_ (Kermel, Tianjin Kermel Chemical Regent Co. Ltd), catechol (Macklin, Shanghai Macklin Biochemical Technology Co. Ltd), guaiacol (Aladdin, Shanghai Aladdin Biochemical Technology Co. Ltd), H_2_O_2_ (Lircon, Shan Dong Lircon Medical Technology Co. Ltd), BaCl_2_ (Kermel, Tianjin Kermel Chemical Regent Co. Ltd), NaOH (Guangfu, Tianjin Guangfu Technology Development Co. Ltd), oxalic acid (Guangfu, Tianjin Guangfu Technology Development Co. Ltd), and phenolphthalein (Guangfu, Tianjin Guangfu Technology Development Co. Ltd).

### 2.2 Treatments

Fresh‐cut potato slices were randomly split into five groups. Each group was immersed in 0.3 mmol L^−1^ ascorbic acid, 0.7 mmol L^−1^ L‐cysteine, 2.0 mmol L^−1^ sodium nitroprusside, 0.7 mmol L^−1^ sodium hydrosulfide, or distilled water (controls) at room temperature for 10 min. After immersion, the samples were centrifuged to dry, put on a plastic plate, covered with a polyethylene film (Miaojie brand, Tupu Daily Chemicals Co. Ltd), and finally stored at 4°C in darkness for 6 days. The experimental measurements were done every 2 days. Five fresh‐cut potato slices per plate and three plates per treatment were involved in the following parameter assessments at each measurement time. Generally, fresh‐cut potato slices' browning, weight loss, rot index, and respiration rate were measured first. The samples were then frozen in liquid nitrogen for 3–5 min, ground into powder by a mill (CG‐9023, Long Plus, Long‐plus Electric Appliances Co. Ltd), and stored at −80°C for further analysis on total phenolic content and enzyme activities. The experiment was repeated twice.

### 2.3 Parameter assessments

#### 2.3.1 Browning

The L* value of fresh‐cut potato slices represents brightness and can be reflected as browning (Hunjek et al., 2020; Tsouvaltzis et al., 2011). The L* of five fresh‐cut potato slices was measured by Colorimeter (NH310+, Sanenshi Intelligent Technology Co. Ltd) individually. An average of 5 values was used per replicate and three replicates per treatment were involved in this assessment.

#### 2.3.2 Total phenolics content

The tissue sample was collected from five fresh‐cut potato slices and the total phenolics content was determined according to the assay of Li et al. (2021) with some modifications. A standard curve of gallic acid was established first. The powder sample (1.0 g) was mixed with 5 mL 80% ethanol and centrifuged at 4°C at 12,000 *g* min^−1^ for 15 min. The supernatant (0.2 mL) was mixed with 1 mL 0.25 mol L^−1^ Folin–Ciocalteu reagent and 3.0 mL 7.5% Na_2_CO_3_. After the mixture was incubated for 1 h at room temperature in the darkness, the absorbance was measured at 765 nm. Three replicates per treatment were involved in this assessment.

#### 2.3.3 PPO and POD activities

The tissue sample was collected from five fresh‐cut potato slices and enzyme (PPO and POD) activities were determined according to the assay of Li et al. (2021) with some modifications. The powder sample (1.0 g) was weighed into a 10 mL centrifuge tube, and 5 mL phosphate buffer (0.1 mol L^−1^, pH 6.5) was added. The mixture was vortexed thoroughly and centrifuged at 12,000 *g* min^−1^ at 4°C for 15 min. The supernatant was collected for PPO and POD activities assay. PPO activity assay: 1.0 mL 0.02 mol L^−1^ catechol, 1.8 mL phosphate buffer (0.1 mol L^−1^, pH 6.5), and 0.2 mL crude enzyme extract were mixed and vortexed thoroughly. The absorbance was measured at 410 nm and recorded every minute. One unit (U) was defined as ∆0.01 in absorbance per minute. Phosphate buffer was used as a control instead of crude enzyme solution and the determination was repeated three times. POD activity assay: 2.7 mL phosphate buffer (0.1 mol L^−1^, pH 6.5), 0.2 mL 0.05% H_2_O_2_, 0.5 mL 2% guaiacol, and 0.1 mL crude enzyme extract were mixed. The absorbance at 470 nm was recorded every minute. One unit (U) was defined as ∆0.01 in absorbance per minute. Phosphate buffer rather than crude enzyme extract was used as a control. Three replicates per treatment were involved in this assessment.

#### 2.3.4 Rot index

The rotted area of fresh‐cut potato slices was divided into four grades according to Gao et al. (2018). Grade 0 means unrotten; Grade 1 means mild putrefaction, a putrefaction area of less than one‐fourth of the potato slice area; Grade 2 means moderate decay, the rotting area between one‐fourth to half of the potato slice area; and Grade 3 means severe rot, more than half of the potato slice area. The rot index was calculated by the following equation. Five fresh‐cut potato slices per replicate and three replicates per treatment were involved in this assessment.

$$\mathrm{Rot}\;\text{index}\;\left(\%\right)=\frac{\sum \left(\text{grade}\times \text{fruit number of this grade}\right)}{4\times \text{total fruit number}}\times 100\%$$

#### 2.3.5 Respiration rate

The respiration rate was measured according to the method of Wang et al. (2022). Ten milliliters of 0.4 mol L^−1^ NaOH were added to a petri dish, and the petri dish was then placed at the bottom of the dryer. Fresh‐cut potato slices were placed on the separator inside the dryer, and the dryer was sealed for 0.5 h. The lye solution in the Petri dish was then transferred into a beaker. Saturated BaCl_2_ (5 mL) and two drops of phenolphthalein were added into the beaker, mixed thoroughly, and titrated with 0.2 mol L^−1^ oxalic acid until the color changed. No fresh‐cut potato samples were in the dryer in the control group. According to the following equation, the respiration rate of the samples was calculated. Three replicates per treatment were involved in this assessment.

$$\text{Respiration rate}\;\left(\mathrm{mg}\;{\mathrm{kg}}^{-1}\;h^{-1}\right)=\left(V_{1}-V_{2}\right)\times c\times \frac{22}{m\times t}$$

(c: the concentration of oxalic acid, mol L^−1^; *V*_1_: the quantity of oxalic acid in the control group, mL; *V*_2_: the quantity of oxalic acid in the treated group, mL; *m*: sample weight, kg; *t*: reaction time, h).

#### 2.3.6 Weight loss

The weight of five fresh‐cut potato slices was done by a digital scale (XB 220A, Precisa, Precisa Gravimentrics AG). The initial weight was recorded as m_0_, and the weight of the samples after treatment on different days was recorded as m_1_. Weight loss was calculated by the following equation. Three replicates per treatment were involved in this assessment.

$$\text{Weight loss}\;\left(\%\right)=\frac{\left(m_{0}-m_{1}\right)}{m_{0}}\times 100\%$$

### 2.4 Experimental design and statistical analysis

A completely randomized design was used in this experiment. The results were expressed as mean ± standard error. All the data, excluding the rot index data, were analyzed by one‐way ANOVA using IBM Statistics SPSS software 26 (IBM Corporation), and means were compared via Duncan's test at *p* = .05.

## 3 RESULTS

### 3.1 Browning

**FIGURE 1:**
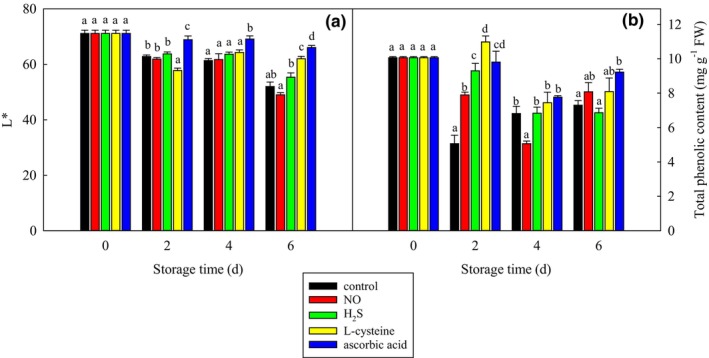
Effect of different food additives on the L* (a) and total phenolic content (b) of fresh‐cut potato slices. The data were expressed as mean ± standard error (*n* = 3). Different letters each day show significant differences at *p* = .05.

The L* value can reflect the browning and the lower L* value indicates the more browning. As shown in Figure 1a, the L* value of all the treatments decreased with the storage time, which means that the browning of all the treatments increased as the storage time increased. The ascorbic acid treatment showed a significantly (*p* < .05) higher L* than the control treatment at any storage time, which means that the ascorbic acid treatment showed significantly (*p* < .05) less browning than the control treatment during the whole storage period. The L‐cysteine treatment showed a significantly (*p* < .05) less L* on day 2 and higher L* on day 6 as compared with the control treatment, which means that the cysteine treatment showed a significantly (*p* < .05) higher browning on day 2 and less browning on day 6 than the control treatment. The NO and H_2_S treatments showed similar L* (browning) as the control treatment at any storage time.

### 3.2 Total phenolic content

As shown in Figure 1b, as the storage time increased, the total phenolic content of the control treatment decreased at first and increased afterward. As compared with the control treatment, ascorbic acid increased total phenolic content by 94% and 26% on days 2 and 6, respectively. On day 2, L‐cysteine and H_2_S increased total phenolic content by 117% and 84%, respectively. These two treatments showed similar levels as the control treatment afterward. NO increased total phenolic content by 56% on day 2, decreased by 26% on day 4, and showed a similar level as the control treatment on day 6.

### 3.3 PPO and POD activities

**FIGURE 2:**
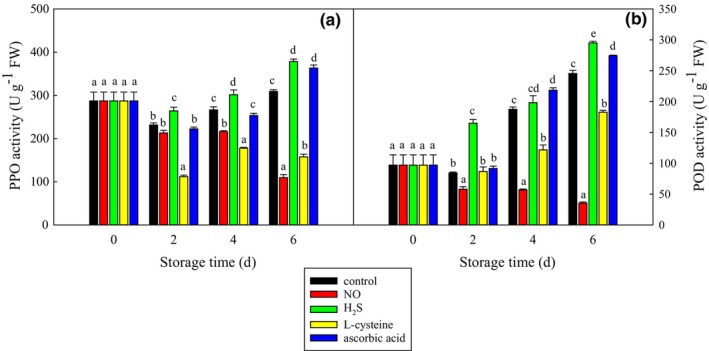
Effect of different food additives on polyphenol oxidase (PPO) (a) and peroxidase (POD) (b) activities of fresh‐cut potato slices. The data were expressed as mean ± standard error (*n* = 3). Different letters each day show significant differences at *p* = .05.

Figure 2a shows that the PPO activity generally decreased at first and increased during storage. The ascorbic acid treatment showed a significantly (*p* < .05) higher PPO activity than the control treatment until day 6. The L‐cysteine treatment showed significantly (*p* < .05) lower PPO activity than the control treatment since day 2, while the H_2_S treatment showed significantly (*p* < .05) higher PPO activity than the control treatment. The NO treatment showed a similar PPO activity on day 2, and a lower PPO activity on days 4 and 6 as compared to the control treatment.

As shown in Figure 2b, the POD activity generally increased with storage time except for the NO treatment. On days 4 and 6, the ascorbic acid treatment showed significantly (*p* < .05) higher POD activity than the control treatment, but the cysteine treatment showed significantly (*p* < .05) lower POD activity. The H_2_S treatment showed significantly (*p* < .05) higher POD activity than the control treatment during the whole storage. However, the NO treatment showed significantly (*p* < .05) lower POD activity than the control treatment.

### 3.4 Rot index

**FIGURE 3:**
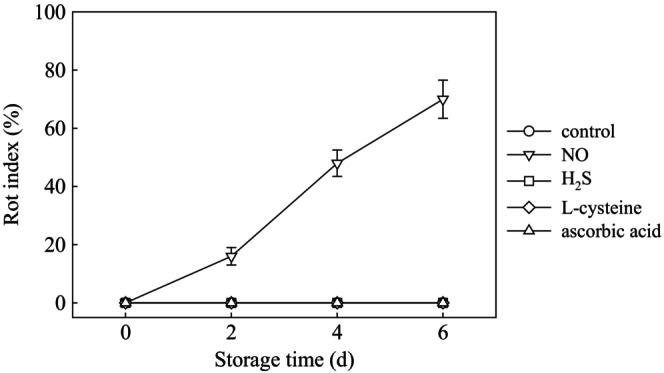
Effect of different food additives on the rot index of fresh‐cut potato slices. The data were expressed as mean ± standard error (*n* = 3). Different letters show significant differences at *p* = .05.

As shown in Figure 3, the H_2_S, L‐cysteine, and ascorbic acid treatments did not show any rot during storage. However, the NO treatment showed a significantly higher rot index than the control treatment on day 2 and the rot index increased afterward.

### 3.5 Respiration rate

**FIGURE 4:**
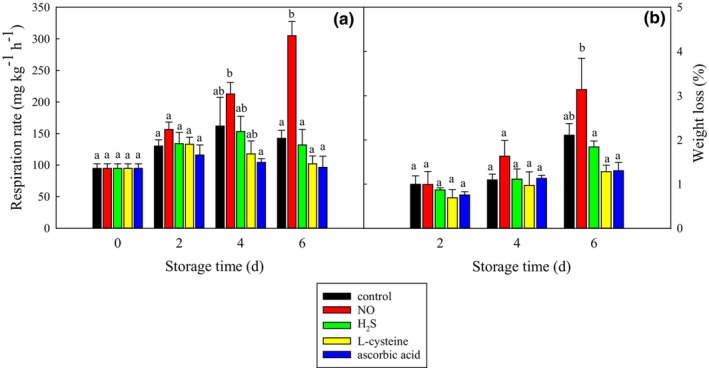
Effect of different food additives on the respiration rate (a) and weight loss (b) of fresh‐cut potato slices. The data were expressed as mean ± standard error (*n* = 3). Different letters each day show significant differences at *p* = .05.

As shown in Figure 4a, the respiration rate of the control group increased at first and decreased afterward. The NO showed a similar respiration rate as the control treatment on days 2 and 4 and increase in the respiration rate by 110% on day 6. The other treatments showed similar levels of respiration rate as the control group.

### 3.6 Weight loss

As shown in Figure 4b, the weight loss of fresh‐cut potato slices increased with storage time. No significant differences were between any treatment and control at any storage time. However, the L‐cysteine treatment showed a significantly (*p* < .05) higher weight loss than the ascorbic acid, H_2_S, and NO treatments on day 6.

## 4 DISCUSSION AND CONCLUSION

Surface browning is the main factor influencing the commercial value of fresh‐cut potatoes (Erihemu et al., 2021). The present study showed that L‐cysteine (0.7 mmol L^−1^) and 0.3 mmol L^−1^ of ascorbic acid reduced fresh‐cut potato browning, while H_2_S (0.7 mmol L^−1^) and 2 mmol L^−1^ of NO did not. A similar phenomenon was found in the repeated experiment. We speculated that the concentration of H_2_S and NO commonly used is not effective for controlling fresh‐cut potato browning, or that these two signal molecules have no inhibiting effects on the browning.

Fresh‐cut potato browning is generally known as enzymatic browning. Ascorbic acid inhibits the enzymatic browning by lowering pH resulting in a decrease in enzyme (PPO and POD) activities (Li et al., 2023), or converting oxidized phenolic compounds back to their original form (Tang et al., 2023), or activating phenylpropanoid metabolism to increase total phenolic content (Zhou et al., 2021). In this study, ascorbic acid (0.3 mmol L^−1^) increased total phenolic content, and PPO and POD activities. It is suggested herein that ascorbic acid can inhibit fresh‐cut potato slices' browning mainly by converting oxidized phenolic compounds back to their original form and possibly by activating phenylpropanoid metabolism. More research on phenolic types and their concentration will help understand the mechanism.

L‐cysteine (0.7 mmol L^−1^) decreased PPO and POD activity, which agrees with the finding that L‐cysteine reduces PPO and POD activities to decrease browning disorders in fresh‐cut vegetables and fruits (Erihemu et al., 2022; Jia et al., 2023). Interestingly, the L‐cysteine treatment showed higher browning on day 2 and lower browning on day 6 than the control treatment. It may be because enzymes oxidize phenolic compounds to be quinones first, and then L‐cysteine reacts with the quinones to give colorless adducts (Ali et al., 2016).

Generally, H_2_S (0.7 mmol L^−1^) and NO (2.0 mmol L^−1^) inhibit the browning of fresh‐cut fruits and vegetables by reducing PPO and POD activities or regulating phenolic metabolism or activating defensive enzyme system (Huque et al., 2013; Sun et al., 2015). However, the present study showed that H_2_S (0.7 mmol L^−1^) failed to inhibit the browning of fresh‐cut potato slices, reduce PPO and POD activities, or influence total phenolic content. Although NO (2 mmol L^−1^) decreased PPO and POD activities, it did not reduce the browning of fresh‐cut potato slices either. Instead, it caused a higher rot index and respiration rate, possibly because the concentration of NO is too high for fresh‐cut potato slices (Zhu et al., 2005). Weight loss is mainly due to respiration and transpiration (Zhao et al., 2020), and NO is expected to increase weight loss in Figure 4b. The other agents, such as H_2_S, cysteine, and ascorbic acid, were found not to influence weight loss, respiration rate, or rot index.

In summary, L‐cysteine (0.7 mmol L^−1^) and ascorbic acid (0.3 mmol L^−1^) reduce the browning of fresh‐cut potato slices without affecting weight loss, respiration rate, or rot index. Ascorbic acid with a concentration of 0.3 mmol L^−1^ inhibits the browning of fresh‐cut potato slices mainly by reducing quinones back to phenolic compounds. L‐cysteine with a concentration of 0.7 mmol L^−1^ reduces the browning mainly by decreasing PPO and POD activities. Nonetheless, these proposed mechanisms need to be studied further.

## AUTHOR CONTRIBUTIONS

**Guoqin Li:** Conceptualization (equal); data curation (equal); formal analysis (lead); methodology (lead); writing – original draft (lead); writing – review and editing (equal). **Xinxin Wang:** Data curation (equal); writing – original draft (supporting). **Hongmei Zhu:** Conceptualization (equal); writing – review and editing (equal). **Guifeng Li:** Conceptualization (equal); writing – review and editing (equal). **Junjie Du:** Conceptualization (equal); writing – review and editing (equal). **Xiaoqing Song:** Conceptualization (supporting); writing – review and editing (equal). **Erihemu:** Conceptualization (equal); methodology (supporting); supervision (lead); writing – review and editing (equal).

## FUNDING INFORMATION

This research work was supported by a Grant from The Foundation (2021DCXM87) of Shanxi Normal University, The Fundamental Research Program of Shanxi Province (20210302124515, 20210302123334, 20210302124263), The Teaching Reform Program of Shanxi Province (J20221509, J20221512), and National Natural Science Foundation Project of Shanxi Normal University (JCYJ2022026).

## CONFLICT OF INTEREST STATEMENT

The authors declare that they have no conflict of interest.

## Data Availability

The data supporting this study's findings are available from the corresponding author upon reasonable request.
